# Closed reduction and intramedullary nails for acute completely displaced femoral diaphysis fracture in children aged 2–6

**DOI:** 10.3389/fped.2024.1346456

**Published:** 2024-04-11

**Authors:** Fei Qiao, Xinpeng Shang, Fei Jiang

**Affiliations:** Department of Pediatric Orthopaedic, Dalian Women and Children’s Medical Group, Dalian, Liaoning, China

**Keywords:** femoral fracture, children, elastic stable intramedullary nailing, closed reduction, fixation

## Abstract

**Background:**

The management of femoral fractures in children aged two to six years is still controversial. The purpose of this study was to assess the results of closed reduction and elastic stable intramedullary nail (ESIN) fixation in completely displaced fractures of the femoral diaphysis in children in this age group.

**Methods:**

A retrospective review of all children with acute completely displaced fractures of the femoral diaphysis in children aged 2–6 years treated from 2013 to 2020 was performed. A total of 34 patients were treated who met the inclusion criteria: Group 1: 21 fractures (transverse and short oblique); Group 2: 13 fractures (long oblique and spiral) that underwent closed reduction and elastic stable intramedullary nail (ESIN) fixation. No differences existed between the 2 groups with respect to age, extremity, sex, time to treatment, mechanism of injury, or fracture displacement. Demographic characteristics and radiographs were reviewed, and the following parameters were documented: surgery time, time to union, return to activities, range of motion of knee joints, and complications. Major complications were defined as those with presumptive long-term side effects or those requiring a reoperation. No major complications were observed in the two groups. All included fractures were treated by a single senior paediatric surgeon. The mean follow-up period was 28.4 months (range 24–45 months). The level of significance was set at *p* < 0.05.

**Results:**

Thirty-four children with acute completely displaced fractures of the femoral diaphysis were included: Group 1: 21 fractures; Group 2: 13 fractures. The patients included 15 girls (44.1%) and 19 boys (55.9%), with an average age of 4.4 years (range 2.8 to 6.5 years). The mean follow-up period was 28.4 months (range 24.2–45.0 months). The demographic characteristics did not differ between the two groups of patients. Overall, successful closed reduction and elastic stable intramedullary nail (ESIN) fixation could be achieved in all 34 patients. The mean surgical time was 40.4 and 43.0 min in Group 1 and Group 2, respectively (*p* = 0.857). Fluoroscopy time was not significantly different between the two groups (37.0 vs. 36.1 s, respectively; *p* = 0.247). Cosmetic results were described as good and satisfactory by all patients. There were no refractures and no incidences of nonunion or growth arrest in the proximal epiphysis. Only two patients suffered from a superficial infection, which was resolved after the pins were shortened and oral antibiotics were administered.

**Conclusion:**

Closed reduction and elastic stable intramedullary nail (ESIN) fixation can be successfully used to treat completely displaced fractures of the femoral diaphysis in children aged two to six years. This technique is efficient and minimally invasive, and the results are satisfactory.

## Background

Fractures of the femoral diaphysis comprise approximately 4% of all long-bone fractures in children and are the second most frequent localization affecting the lower extremities (1–4). Historically, paediatric diaphyseal fractures of the femur have been treated conservatively with a hip spica cast for the femur and traction (4–10). More recently, a trend towards aggressive treatment of these fractures in patients of different ages has been observed: elastic stable intramedullary nailing (ESIN) for school-aged children (11) or even adolescents (12, 13); rigid intramedullary nails for adolescents; and external fixation for complex fractures or for children suffering multiple severe injuries (14) to allow for easier mobilization and quicker recovery while decreasing the risk of late deformity.

Since the late 1980s, ESIN has been reported to be safe and effective in treating diaphyseal fractures of the femur in school-age children (15–24), but in the case of younger children, there is no consensus and continuing debate between those surgeons preferring nonoperative methods and those supporting a more aggressive approach such as ESIN (25, 26). Recently, several authors have focused on examining the results of extending flexible intramedullary stabilization into the preschool age group (25, 27–29). The guidelines of the German Society of Paediatric Surgery (30) recommend that femoral shaft fractures be treated by ESIN in children older than 2–3 years of age to avoid complications and economic burdens on families. Convenience with transport, earlier motion, no secondary displacement and decreased burden on the patients' care provider are among the potential advantages of ESIN.

The purpose of this study was to evaluate the effect of treating femoral fractures in children aged two to six years using closed reduction and ESIN. We investigated whether the use of ESIN would yield good results for acute completely displaced fractures of the femoral diaphysis.

## Methods

### Patients

This study was approved by the Institutional Ethical Review Board of Dalian Women and Children's Medical Group (approval number DLET-KY-2022-03). Written informed consent was obtained from all guardians for anonymized data analysis and publication. A total of 36 patients with acute completely displaced fractures of the femoral diaphysis were treated at our hospital from October 2013 to September 2020. Patients with a confirmed diagnosis of acute completely displaced fractures of the femoral diaphysis were divided into two groups according to the type of fractures, fracture: Group 1 included 21 fractures (transverse and short oblique), and Group 2 included 13 fractures (long oblique and spiral).

The inclusion criteria were as follows: (1) patients with a confirmed diagnosis of acute completely displaced fractures of the femoral diaphysis; (2) treatment [closed reduction and elastic stable intramedullary nail (ESIN) fixation] at our institution within 48 h of injury; (3) standard preoperative anteroposterior (AP) and lateral injured leg radiographs; (4) follow-up duration > 18 months; and (5) complete clinical and radiographic data.

The exclusion criteria were as follows: (1) poly-traumatized patients with other associated fractures; (2) open, comminuted or pathological fractures; (3) follow-up less than 18 months; and (4) incomplete clinical and radiographic data.

A total of 34 of 36 patients were followed up for a mean of 28.4 months (range 24.2–45.0 months). All surgeries were performed by a single senior paediatric surgeon, and the average surgery time was 41.4 min (range 25.0–50.0 min). The injured leg was immobilized with a single leg-spica brace for 4 weeks and evaluated radiologically and clinically.

### Surgical procedures

**Figure 1:**
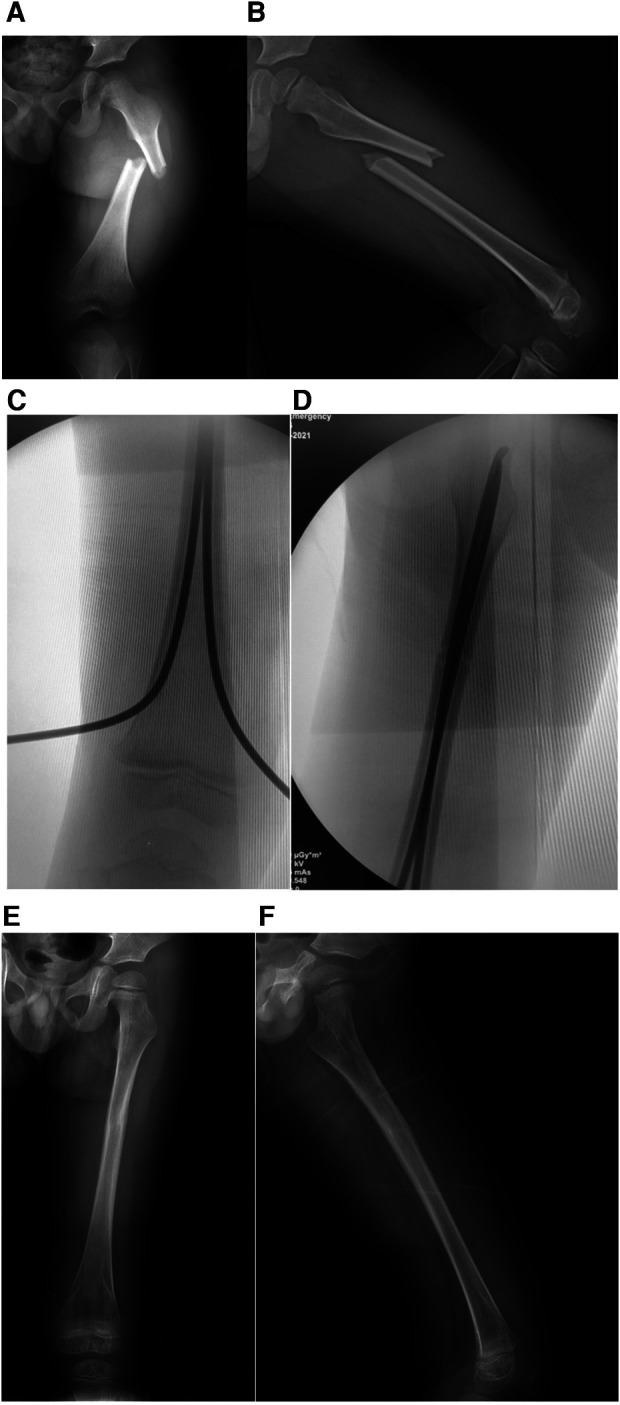
Typical short oblique fracture of left femur of a 4 years old boy. (**A**) The initial AP x-ray of left femur preoperative. (**B**) lateral x-ray of left femur preoperative. (**C**) An entry point was made with the help of a bone awl approximately 2.0–3.0 cm above the physis on the lateral side. (**D**) C-arm result after closed reduction and ESIN fixation. (**E**) 10 months follow-up AP x-ray of left femur. (**F**) 10 months follow-up lateral x-ray of left femur.

**Figure 2:**
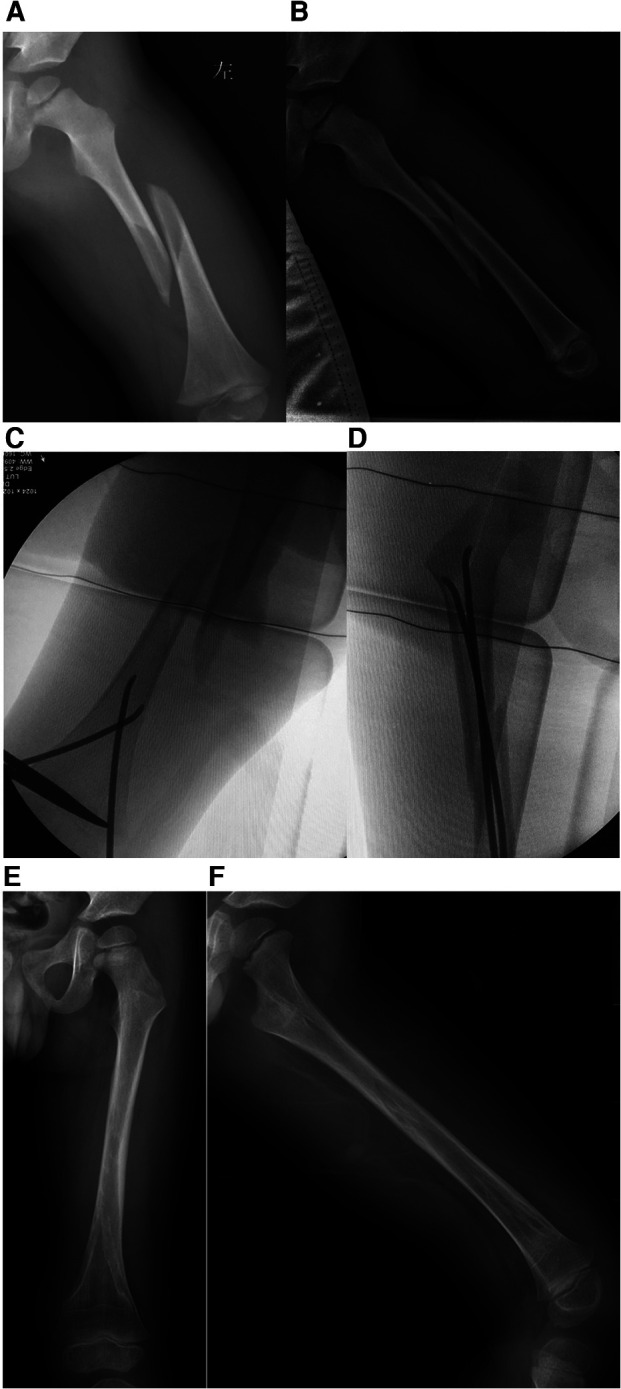
Typical long spiral fracture of left femur of a 3 years old boy. (**A**) The initial AP x-ray of left femur preoperative. (**B**) lateral x-ray of left femur preoperative. (**C**) An entry point was made with the help of a bone awl approximately 2.0–3.0 cm above the physis on the lateral side. (**D**) C-arm result after closed reduction and ESIN fixation. (**E**) 9 months follow-up AP x-ray of left femur. (**F**) 9 months follow-up lateral x-ray of left femur.

The patients were placed on an orthopaedic fracture table, general anaesthesia was administered, the skin was prepared, and reduction of the fracture by traction guided by fluoroscopy was performed (Figures 1A,B, 2A,B). The size of the ESIN is determined by the canal diameter at the isthmus of the femur on the AP x-ray, and then, the ESIN size is selected to be ∼40% of that measurement to obtain at least 80% canal fill (4, 15, 16). Nails preangled, as described by Ligier et al. (15), at 45° approximately 2 cm from one end were used. An entry point was made approximately 2.0–3.0 cm above the physis on the lateral side with the help of a bone awl. A nail was then inserted through the entry point into the medullary canal by rotator movements of the wrist and advanced up to the fracture site. Another nail was introduced using the same technique from the medial side and advanced up to the fracture site. The nails were then placed across the already reduced fracture site one by one. Fluoroscopy was used to ensure that both nails were in the canal across the fracture site. The traction was released when the nails had crossed the fracture site, after which the nails were advanced further. The medial nail was advanced until it was within 2 cm of the proximal femoral capital physis, whereas the lateral nail was inserted until it was approximately 1 cm from the greater trochanteric physis. The nails were left in a protruding position approximately 0.5–1.0 cm from the distal end to ensure that they could be easily removal later in the healing process (Figures 1C,D, 2C,D) (31).

Postoperatively, patients remained in the hospital for pain control; generally, within 2 days, they were deemed comfortable enough to be discharged home on oral analgesia. During the postoperative period, the limb was immobilized with a single-leg hip spica brace for 4 weeks based on the preoperative fracture pattern and fracture healing to reduce postoperative pain and facilitate home care. Then, the patients were encouraged to perform exercises with active, active-assisted and passive knee range of motion. Weight bearing status depended on the fracture configuration after the bridging callus appeared, but in general, partial weight bearing was started at approximately 6 weeks and progressed to full weight bearing when the fracture line was not visible on AP and lateral x-rays.

### Postoperative evaluation

Then, fixation and bone union were radiographically examined on patients scheduled for postoperative follow-up visits at two weeks, four weeks, six weeks, three months and six months postoperatively and thereafter at half-year intervals. Bone union was indicated by the disappearance of the fracture lines in 3 of the 4 cortices on both the AP and lateral radiographs of the fracture. Delayed healing was characterized by a lack of solid bone union on both the AP and lateral radiographs six months after surgery. At each follow-up, the patients were assessed radiologically and clinically. The nails were removed when complete healing of the fracture occurred (usually between 8 and 10 months). The final results were evaluated using the criteria of Flynn et al.

### Statistical analysis

SPSS v22 (IBM Corp., Armonk, NY, USA) was used for statistical analysis. Categorical data were compared with the *χ*^2^-test. For nonnormally distributed data, the Mann‒Whitney *U*-test for independent samples was conducted. The differences in the continuous data between the two groups were compared with independent-samples *t*-tests. The level of significance was set to *p* < 0.05.

## Results

Thirty-four patients who met the inclusion criteria were treated at our institution during the study period. The average patient age at the time of injury was 4.4 years (range, 2.8–6.5), and the mean length of follow-up was 28.4 months (range 24.2–45.0 months), Group 1 included 21 (61.8%) fractures (transverse and short oblique), and Group 2 included 13 (38.2%) fractures (long oblique and spiral). The baseline characteristics did not differ between the two groups of patients. The fractures were equally divided between the left and right sides. All of the fractures were closed and reduced, and all were neurovascularly intact. The mean surgical time was 40.4 and 43.0 min in Group 1 and Group 2, respectively (*p* = 0.857). The fluoroscopy time was not significantly different between the two groups (37.0 vs. 36.1 s, respectively; *p* = 0.247). The clinical outcome according to the criteria of Flynn et al. (22) was excellent in 19 out of 21 (90.5%) patients in Group 1 and good in the remaining two patients. A similar outcome was found in patients in Group 2, with excellent in 12 out of 13 (92.3%) patients and good in the remaining 1 patient. The overall complication rate was 19.0% (4/21) in Group 1 and 23.1% (3/13) in Group 2 (*p* = 0.781). Four complications were noted in Group 1: three cases of pin site infection. Three complications were noted in Group 2: two cases of pin site infection. There were no refractures or incidences of nonunion, growth arrest in the distal epiphysis, or limb length discrepancy within 1.0 cm in either group (Table 1) (Figures 1E,F, 2E,F).

**Table 1:** The demographic characteristics, evaluation results and complications of the patients and fractures.

	Group 1 (*n* = 21)	Group 2 (*n *= 13)	*p-*value
Age			
Mean age (years)	4.5	4.2	0.082
Sex, *n* (%)			
Female	9	6	0.889
Male	12	7	
Side of fracture			
Right	10	7	0.471
Left	11	6	
Surgery time (min)	40.4	43.0	0.857
Complications (%)	19.0% (4/21)	23.1% (3/13)	0.781
Union of fracture (weeks)	4.5	4.8	0.071
limb length discrepancy (cm)	0.41	0.37	0.728
Fluoroscopy time (s)	37.0	36.1	0.247

Statistically significance was set to be *p* < 0.05.

## Discussion

Our study shows that acute completely displaced fractures of the femoral diaphysis in children aged 2–6 can successfully be treated by closed reduction and ESIN fixation with good functional outcomes; only a few cases of pin site infection were detected among the patients. These problems, due to prominent hardware at the nail insertion site, were resolved within a few days by giving oral antibiotics and analgesics. In addition, there were equal operating times and radiation exposure in the two groups. There was no clinic-radiological difference in terms of time to fracture site union and full weight bearing between the two groups. The range of motion at the hip and knee was normal in all patients after the removal of the ESINs. There were no patients with angulation deformities in the sagittal plane or coronal plane in either group.

Transverse and short oblique femoral shaft fractures in children have been treated by closed reduction and ESIN fixation in the past based on good results and fewer complications. In recent years, surgeons have preferred these operative techniques for long oblique and spiral fractures of the femoral shaft; therefore, the majority of paediatric femoral shaft fractures are now treated operatively. Families benefit more from shorter hospital stays and less economic burden and complications than from conservative treatment, such as submuscular plating or external fixation. ESIN is often recommended as the best treatment for children aged 6–12 years, ideally weighing less than 50 kg (13, 20, 32–34). There are no previous comparative studies between Group 1 and Group 2 femoral shaft fractures in preschool-aged patients. In our study, the number of patients in each group with a normal range of motion at the hip and knee joints was the same at the final follow-up. Similar results were obtained by the studies of Khazzam et al. (35), Gyaneshwar et al. (36) and Lohiya et al. (37). The average duration of progression to full weight bearing in our study was 10.4 weeks in Group 1 and 11.0 weeks in Group 2 (*p* = 0.479). In the study by Lohiya et al. 10 (37), the mean duration to full weight bearing was 10.5 weeks.

In our research, minor complications were observed in 19.0% of Group 1 and 23.0% of Group 2 with no major complications. In the study by Gyaneshwar et al. (36), the minor complication rate was significantly higher, 47.06% of patients in the titanium group and 35.29% of patients in the stainless steel group. Fewer major complications were found. There was no significant difference in the malunion deformity rate in either group in the sagittal and coronal planes. Wall et al. (38) reported that the malunion rate in the titanium group was 23.2% (13/56), which was much higher than our report. This difference may be because their study did not include the same age group as ours. Moroz et al. and Ho et al. reported that patients older than 10 years of age who underwent ESIN for femoral shaft fractures showed a higher complication rate than younger patients (13, 24). Canavese F et al. also found that a higher rate of complications was observed in patients aged 13 years or older (39). All of the patients in our series were younger than 6 years and had a low rate of complications. We recommend two surgical procedures to reduce minor complications: first, the diameter of the selected ESINs must be as large as possible to fill at least 80%, or even nearly 100% of the canal, to ensure the reliability of fixation; second, after pin cutting, the surgeon should ensure that approximately 0.5–1.0 cm is located at the distal end of the ESINs, which cannot be palpated on the shin.

ESINs prebent in a double C-type configuration with a degree three times the diameter of the intramedullary canal represent the best treatment for transverse fractures in a diaphyseal long bone fracture. Theoretically, the canal diameter at the isthmus should be measured, and 2 equally sized ESINs should be selected to fill at least 80% of the canal of the isthmus (4, 15, 16). Two prebent “C”-shaped nails are generally thought to be an essential part of the ESIN technique in Group 1 fractures, but no evidence for this is available for Group 2 fractures, including long oblique and spiral fractures. Kaise et al. (40) demonstrated *in vitro* that prebent ESINs are important in providing stability in spiral femoral shaft fractures but that the degree of prebending needs to be >30°. Kaiser et al. (41) also recommended the use of a 3rd nail in ESIN in paediatric femur fractures to improve the stability of the osteosynthesis and to reduce peri- and postoperative complications, especially in long oblique and spiral fractures. This is the first mention of the idea of “stacking” the canal. Busch et al. (42) advised that treatment with four ESINs should be considered for skeletally immature patients presenting with length-unstable femur fractures. The concept of “stacking” the femoral canal is mentioned again.

In our Group 2, all patients were treated by two traditional “C shaped” techniques, with ESINs 2.0–3.0 mm in diameter. None of the 13 patients needed a 3rd or 4th ESIN or spica cast, and all patients recovered satisfactorily. In general, there are two reasons for this: first, these patients are younger than the children included in the Kaise et al. study (40). Similarly, the diameter of their canals is limited, and double equally sized ESINs, which are selected to fill more than 80% of the canal of the isthmus, already result in sufficient stiffness and resistance to rotation and axial loading; second, fracture instability leads to malunion due to shortening, varus or recurvatum, and the retrograde 2C configuration creates a construct that utilizes 6 points of intramedullary contact to create stable fixation (15).

Some researchers reported that children treated with ESIN for femoral shaft spiral fractures required further surgery for either unacceptable varus deformity, shortening, or insufficient stability (43). Other authors have commented on the unsatisfactory fixation obtained by ESIN alone in spiral or other complex femoral fractures; Kraus et al. (44) reported external fixation for these fractures, and Sink et al. (45) preferred submuscular plating. In our study, the total outcome according to Flynn's criteria (22) was excellent in 91.2% of patients and satisfactory in 8.8%, with no poor results. However, the clinico-radiological results were not significantly different between the two groups at the final follow-up.

Spica casting and traction are still the recommended treatments for children in their first and second years. In the third year, children are managed nonoperatively and operatively; nevertheless, there is still no strong evidence of a preferred method for surgically treated children. However, ESIN is a widely accepted treatment for femoral shaft fractures in children older than 3 years (4, 5, 10, 28–30, 46). Several studies have reported the safety and benefits of ESIN for preschool femur fractures, including ease of transporting children, fewer minor complications and decreased economy burdens (25, 28, 29, 45, 47). These disadvantages were less common in our study. We confirmed that treatment with ESIN resulted in similar outcomes to those when spica casts and traction are used for treating these fractures in young children with fewer complications.

The analysis of our results showed some limitations. First, to the best of our knowledge, this is the first retrospective study, with a relatively low number of patients, that has compared the clinical and radiological outcomes of femoral shaft fractures in in both Group 1 and Group 2 treated by closed reduction and ESIN fixation in preschool-age children. The retrospective nature of our study is prone to selection and observational biases. Second, it represents a single surgeon's experience. Third, future prospective cohort research would be useful to explore these variables and better define the role of ESIN fixation in length-unstable femur fractures in these children.

## Conclusions

Treatment with a retrograde 2C configuration of ESINs should be considered for skeletally immature patients presenting with length-unstable femur fractures. In our series, all femur fractures treated with these methods achieved union with no significant complications or hardware failure. As a consequence, we recommend that young children with femoral diaphyseal length-unstable fractures with an open physis be treated with this ESIN technique.

## Data Availability

The raw data supporting the conclusions of this article will be made available by the authors, without undue reservation.
